# Construction of arbitrarily strong amplifiers of natural selection using evolutionary graph theory

**DOI:** 10.1038/s42003-018-0078-7

**Published:** 2018-06-14

**Authors:** Andreas Pavlogiannis, Josef Tkadlec, Krishnendu Chatterjee, Martin A. Nowak

**Affiliations:** 1 IST Austria, A-3400 Klosterneuburg, Austria; 2 Program for Evolutionary Dynamics, Department of Organismic and Evolutionary Biology, Department of Mathematics, Harvard University, Cambridge, MA 02138 USA

## Abstract

Because of the intrinsic randomness of the evolutionary process, a mutant with a fitness advantage has some chance to be selected but no certainty. Any experiment that searches for advantageous mutants will lose many of them due to random drift. It is therefore of great interest to find population structures that improve the odds of advantageous mutants. Such structures are called amplifiers of natural selection: they increase the probability that advantageous mutants are selected. Arbitrarily strong amplifiers guarantee the selection of advantageous mutants, even for very small fitness advantage. Despite intensive research over the past decade, arbitrarily strong amplifiers have remained rare. Here we show how to construct a large variety of them. Our amplifiers are so simple that they could be useful in biotechnology, when optimizing biological molecules, or as a diagnostic tool, when searching for faster dividing cells or viruses. They could also occur in natural population structures.

## Abstract (web summary)

Andreas Pavlogiannis et al. present an approach for constructing strong amplifiers of natural selection using evolutionary graph theory. They also identify features of population structures that are necessary for amplification and suggest their algorithm could be used to construct amplifiers in vitro.

## Introduction

In the evolutionary process, mutation generates new variants, while selection chooses between mutants that have different reproductive rates. Any new mutant is initially present at very low frequency and can easily be eliminated by random drift. The probability that the lineage of a new mutant eventually takes over the entire population is called the fixation probability. It is a key quantity of evolutionary dynamics and characterizes the rate of evolution^1–5^.

Consider a population, in which at each time step an individual is chosen for reproduction with probability proportional to fitness, and the offspring replaces another individual^6^. In a well-mixed population, each offspring is equally likely to replace any individual. If the new mutant has relative fitness *r*, then its fixation probability is $(1 - 1{\mathrm{/}}r){\mathrm{/}}(1 - 1{\mathrm{/}}r^N)$, where *N* is the population size^5^. For advantageous mutants, which have $r > 1$, the fixation probability converges to 1 − 1/*r* in the limit of large population size.

Population structure can affect evolutionary and ecological dynamics^7–16^. In evolutionary graph theory, the structure of a population is described by a graph^17–24^: each individual occupies a vertex; the edges mark the neighboring sites where a reproducing individual can place an offspring. The edge weights represent the proportional preference to make such a choice. If each neighbor is chosen uniformly at random, then the outgoing edges of every vertex have identical weights. This is modeled by an unweighted graph. A self-loop represents the possibility that an offspring does not migrate but instead replaces its parent^25^. The classical well-mixed population is described by an unweighted, complete graph with self-loops.

In general, the fixation probability depends not only on the graph, but also on the initial placement of the invading mutants^26, 27^. The two most natural cases are the following. First, mutation is independent of reproduction and occurs at all locations at a constant rate per unit time. Thus, mutants arise with equal probability in each location. This is called uniform initialization. Second, mutation happens during reproduction. In this case, mutants are more likely to occur in locations that have a higher turnover. This is called temperature initialization. Our approach also allows us to study any combination of the two cases: some mutants arise spontaneously while others occur during reproduction.

For a wide class of population structures^17^, which include symmetric ones^28^, the fixation probability is the same as for the well-mixed population. A population structure is an amplifier if it exaggerates the fitness difference between the invading mutant and the resident when compared to the well-mixed population^17, 27, 29^. A population structure is an arbitrarily strong amplifier (for brevity hereafter also called “strong amplifier”) if it ensures a fixation probability arbitrarily close to one for any advantageous mutant, *r* > 1. Strong amplifiers can only exist in the limit of large population size.

Numerical studies^30^ suggest that for spontaneously arising mutants and small population size, many unweighted graphs amplify for some values of *r*. But for a large population size, randomly constructed, unweighted graphs do not amplify^31^. Moreover, proven amplifiers for all values of *r* are rare. For spontaneously arising mutants (uniform initialization): (i) the Star has fixation probability of ~1 − 1/*r*^2^ in the limit of large *N*, and is thus an amplifier^17, 32, 33^; (ii) the Superstar (introduced in ref. ^17^, see also ref. ^34^) and the Incubator (introduced in refs. ^35, 36^), which are graphs with unbounded degree, are strong amplifiers. The mathematical proofs of these assertions are intricate^37^.

For mutants that arise during reproduction (temperature initialization), neither the Star nor the Superstar amplify^27^. The Star can be modified with self-loops and edge weights to obtain the Looping Star, which has fixation probability 1 − 1/*r*^2^ in the limit of large *N* both for mutants that arise during reproduction and for mutants that arise spontaneously. The Looping Star is the only known amplifier for both uniform and temperature initialization^27^, but it is not an arbitrarily strong amplifier. In fact, no strong amplifier for temperature initialization had been known.

In this work we resolve several open questions regarding strong amplification under uniform and temperature initialization. First, we show that there exists a vast variety of graphs with self-loops and weighted edges that are arbitrarily strong amplifiers for both uniform and temperature initialization. Moreover, many of those strong amplifiers are structurally simple, therefore they might be realizable in natural or laboratory setting. Second, we show that both self-loops and weighted edges are key features of strong amplification. Namely, we show that without either self-loops or weighted edges, no graph is a strong amplifier under temperature initialization, and no simple graph is a strong amplifier under uniform initialization.

## Results

### Results overview

**Fig. 1:**
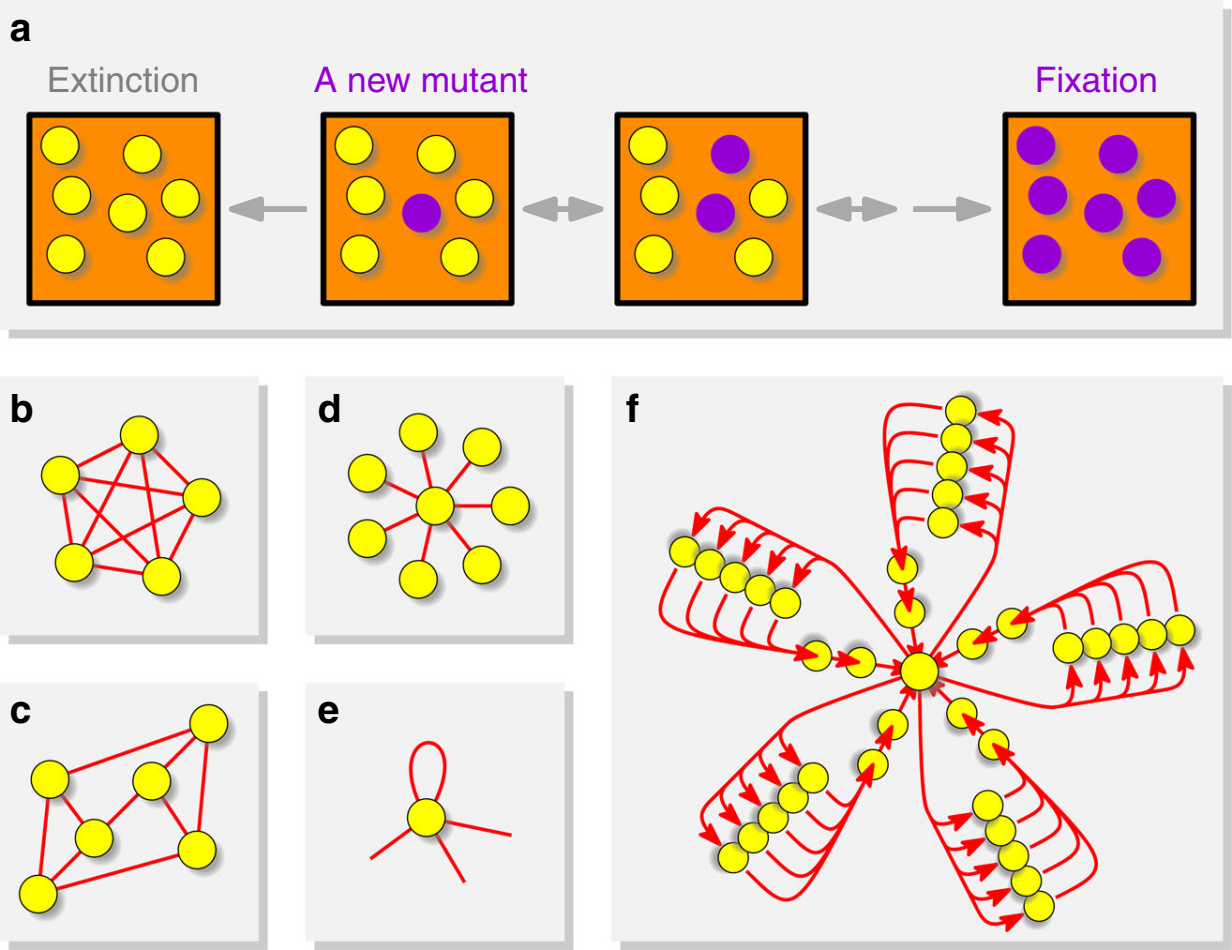
Evolutionary dynamics in structured populations. Residents (yellow) and mutants (purple) differ in their reproductive rate. **a** A single mutant appears. The lineage of the mutant becomes extinct or reaches fixation. The probability that the mutant takes over the population is called “fixation probability”. **b** The classical, well-mixed population is described by a complete graph with self-loops. (Self-loops are not shown here.) (**c**) Isothermal structures do not change the fixation probability compared to the well-mixed population. **d** The Star is an amplifier for uniform initialization. **e** A self-loop means the offspring can replace the parent. Self-loops are a mathematical tool to assign different reproduction rates to different places. **f** The Superstar, which has unbounded degree in the limit of large population size, is a strong amplifier for uniform initialization. Its edges (shown as arrows) are directed which means that the connections are one-way

Our contribution comes in two parts. First, we give an explicit construction of a wide range of strong amplifiers. Second, we identify features of population structures that are necessary for amplification. See Fig. 1 for the illustration of the model and Supplementary Table 1 for the summary of our results.

### Construction of strong amplifiers

We prove that almost all families of connected graphs with self-loops can be turned into arbitrarily strong amplifiers of natural selection by assigning suitable edge weights. The resulting structures are arbitrarily strong amplifiers for both types of mutants: those that arise during reproduction and those that arise spontaneously, or any combination of the two. Our result proves not only the existence of those structures, but provides an explicit procedure for their construction. Note that by assigning small (or even zero) weight to an edge, we can effectively erase it. Hence, our construction is particularly interesting for sparse graphs.

The construction first specifies certain subset of vertices that we call a “hub”. The remaining vertices are split by the hub into a number of short “branches”. The construction guarantees that the combined population size of all the branches is much larger than that of the hub. Therefore, with high probability, the first mutant arises on a branch.

**Fig. 2:**
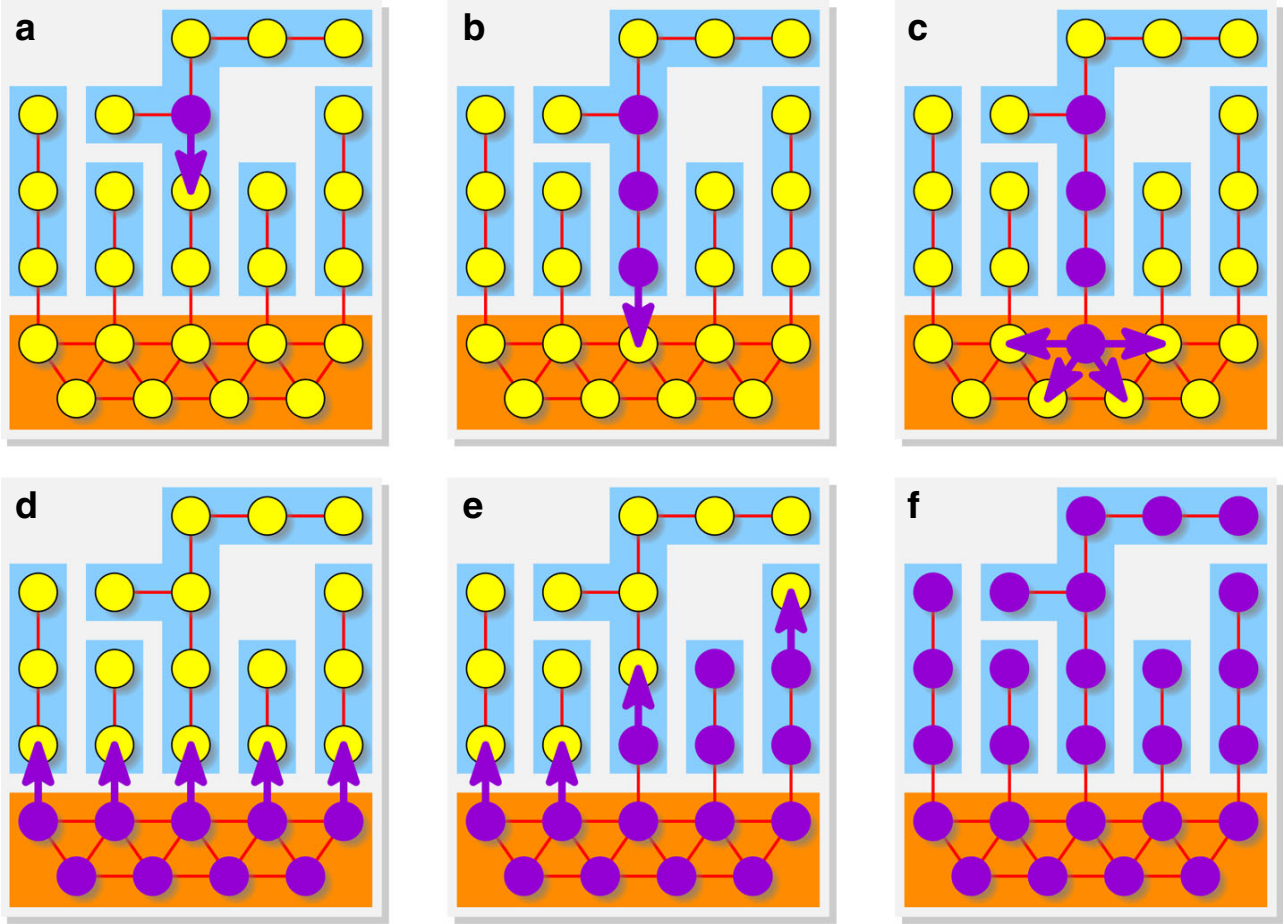
Steps to fixation in strong amplifiers. Residents are shown in yellow, mutants in purple. **a** Our construction partitions a graph into a “hub” (orange) and a number of “branches” (blue) in such a way that the first mutant appears in one of the branches. The fixation of advantageous mutants is then reached in three stages. **b** The mutant lineage reaches the vertex of the branch adjacent to the hub. **c**, **d** Next, the mutants place offspring into the hub several times and eventually fixate there. In the worst case, the initial branch can become all residents again. **e**, **f** Finally, the mutants fixate in the branches one after the other and thereby occupy the whole population

The weights of all edges are then defined so that each of the following steps happens with high probability (Fig. 2). First, the mutants spread on the branch until they reach a vertex that is connected to the hub. Second, the mutants repeatedly invade the hub and eventually fixate there. Third, one by one the mutants spread from the hub to all branches and fixate.

Intuitively, the weight assignment creates a sense of global flow in the branches, directed toward the hub. This guarantees that the first two steps happen with high probability. For the third step, we show that once the mutants fixate in the hub, they are extremely likely to resist all resident invasion attempts and instead they will invade and take over the branches one by one thereby fixating on the whole graph. For more detailed description, see “Methods” section “Construction of strong amplifiers”.

### Simulation results

**Fig. 3:**
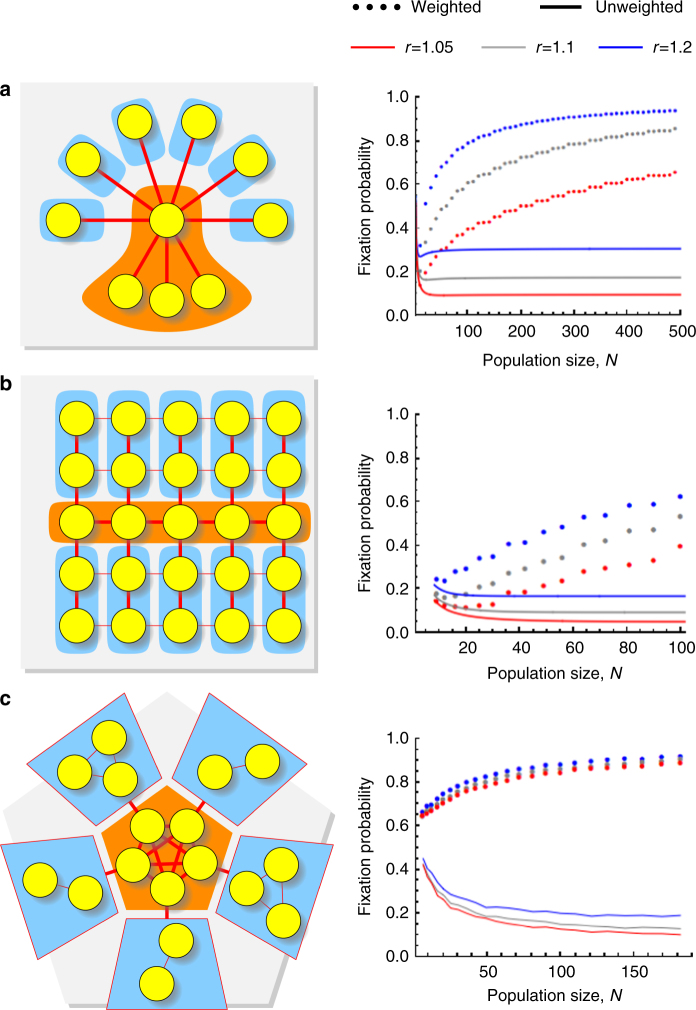
Almost any topology can be turned into a strong amplifier. We illustrate the construction using the topology of a Star (**a**), a Grid (**b**), and a Sunflower (**c**). The hub is shown in orange, the branches in blue. Thin edges are assigned negligibly small (or zero) weights. For each graph, we compare the fixation probability of the unweighted (lines) and the weighted version (dots) as function of the population size, *N* for uniform initialization (for temperature initialization, see Supplementary Figure 2). A Sunflower graph consists of a well-mixed population of size *n* in the center surrounded by *n* petals, which are local well-mixed populations. Each petal is connected to a unique vertex of the center. For details, see Supplementary Note 1, Section 6

Note that simple structures such as Stars, Grids, or Sunflowers can be turned into arbitrarily strong amplifiers using our construction (Fig. 3). The explicit weight construction is described in Supplementary Figure 1. In Fig. 3, we show the results of numerical experiments on the fixation probabilities of advantageous mutants, comparing the weighted structures to their unweighted counterparts. The experiments simulate the evolutionary dynamics on each population structure. We vary the population size and the fitness advantages for the mutant. For each such case, we simulate the evolutionary dynamics many times to obtain an accurate value for the average fixation probability. We observe that although the unweighted structures have small (or no) amplification properties, our construction turns them into strong amplifiers, where advantageous mutants fixate with high probability.

Specifically, in Fig. 3a, we consider Star graphs *S*_*N*_ with *N* = 10, 20, …, 500. For the unweighted Star, there is an exact formula for fixation probability under both uniform and temperature initialization^32^. The values for weighted Star were computed by numerically solving large systems of linear equations.

In Fig. 3b, we consider $n \times n$ and $n \times (n + 1)$ Grid graphs of sizes $N = 9,12,16,20, \ldots ,100$. In order to avoid boundary conditions, the grid “wraps around” that is the vertices in the first row are connected to the vertices in the last row and the same holds for columns. The unweighted Grid with *N* vertices is isothermal so the fixation probability under both uniform and temperature initialization is given by $(1 - 1/r)/(1 - 1/r^N)$.

In Fig. 3c, we consider Sunflower graphs. An *n*-centered Sunflower graph is a graph consisting of a well-mixed population of size *n* in the center and *n* surrounding petals, which are well-mixed population themselves. Each petal is connected with all its vertices to a unique vertex from the center. Specifically, we consider *n*-centered Sunflower graphs where all petals have the same size (either *n* − 1 or *n* − 2). The total population size is $N = 6,9,12,16, \ldots ,182$.

**Fig. 4:**
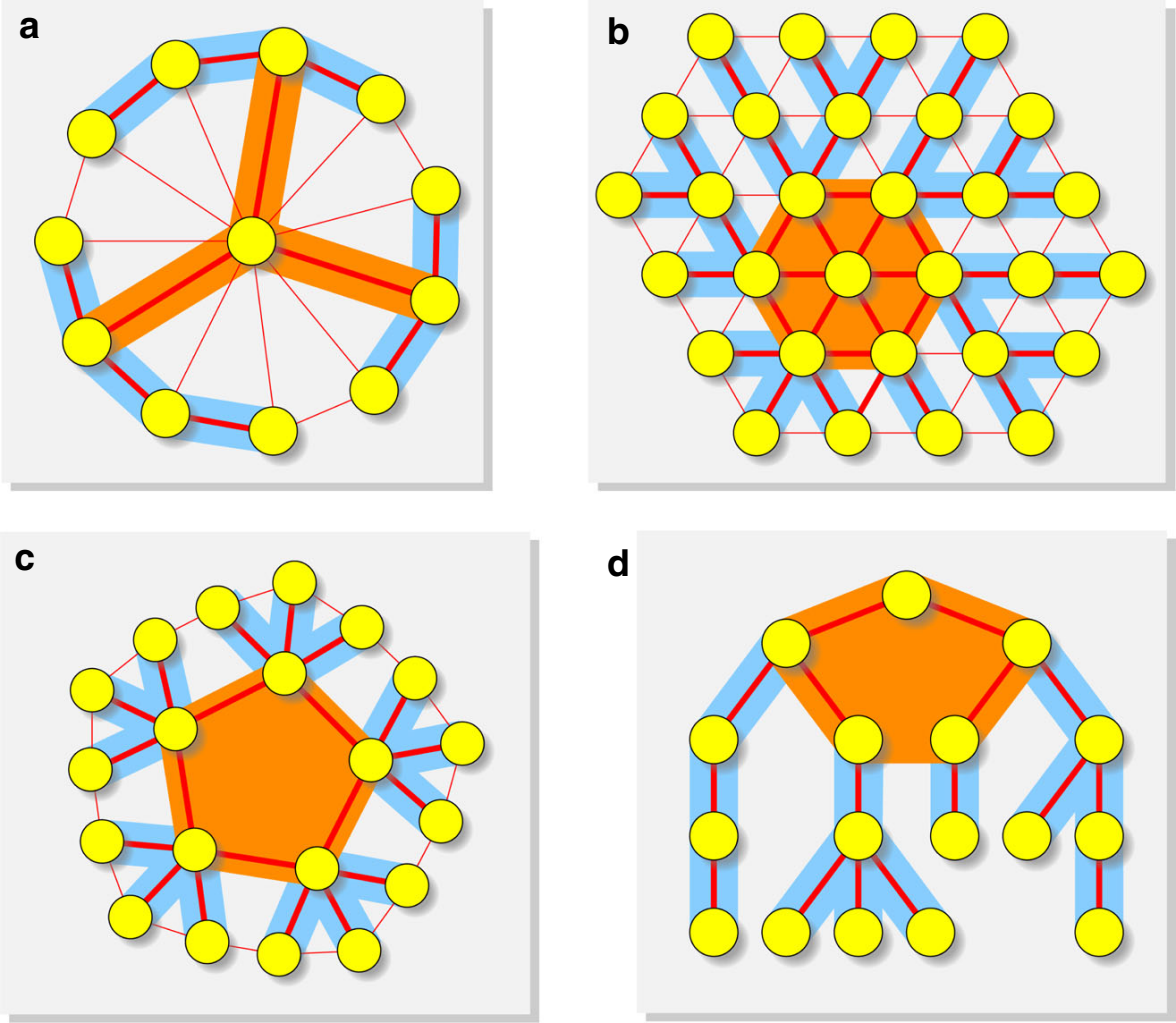
Infinite variety of strong amplifiers. Many topologies can be turned into arbitrarily strong amplifiers (Wheel (**a**), Triangular grid (**b**), Concentric circles (**c**), and Tree (**d**)). Each graph is partitioned into hub (orange) and branches (blue). The weights can be then assigned to the edges so that we obtain arbitrarily strong amplifiers. Thick edges receive large weights, whereas thin edges receive small (or zero) weights

Recall that strong amplifiers can only exist in the limit of large population size. The above simulations illustrate that our weight assignment substantially increases the fixation probability even for graphs with small population size. An illustration of a wide variety of strong amplifiers for different topologies is presented in Fig. 4.

### Necessary conditions for amplification

Our main result shows that a large variety of population structures can provide strong amplification. A natural follow-up question concerns the features of population structures under which amplification can emerge. We complement our main result by proving that both weights and self-loops are essential for strong amplification. Thus, we establish a strong dichotomy. Without either weights or self-loops, no graph can be a strong amplifier under temperature initialization, and no simple graph can be a strong amplifier under uniform initialization. On the other hand, if we allow both weights and self-loops, strong amplification is ubiquitous.

Under temperature initialization, we prove that on every graph without self-loops (but possibly with weighted edges), the fixation probability is at most $1 - 1/(r + 1)$. Similarly, on every graph without weighted edges (but possibly with self-loops), the fixation probability is at most $1 - 1/(4r + 2)$. Hence, if the population structure lacks either self-loops or weights, strong amplification under temperature initialization is impossible.

Under uniform initialization, we prove two analogous results for families of graphs that have bounded degree. Here, a family $\{ G_1,G_2, \ldots \}$ of graphs has bounded degree if there exists a constant *c* such that every vertex of every graph in the family has at most *c* adjacent edges. An example of bounded degree graphs are Grid graphs: every vertex in a rectangular Grid of any size has degree at most 4 (or 8 for Moore neighborhood). We prove that on every graph without self-loops (but possibly with weighted edges), the fixation probability is at most $1 - 1/(c + cr^2)$. Similarly, on every graph without weighted edges (but possibly with self-loops), the fixation probability is at most $1 - 1/(1 + rc)$. It follows that with either of the two restrictions, there exist no strong amplifiers under uniform initialization.

## Discussion

Prior to our finding, strong amplifiers of natural selection have been elusive. Only very few examples of strong amplifiers have been described for spontaneously arising mutants. No strong amplifiers have been known for mutants that arise during reproduction. But here we show that many population structures can be turned into arbitrarily strong amplifiers, for both types of mutants, by choosing suitable edge weights. Consequently, there exists an unlimited variety of population structures that are strong amplifiers of natural selection (Fig. 4). We present an algorithm for their construction. We note that our structures can be remarkably simple.

It is now conceivable that amplifiers of natural selection could be built in the lab. Modern microfluidics technology is capable of building population structures (or, so called “metapopulations”), which control the topology of interactions and migration^38–40^. These metapopulations are typically arranged in microscale patches of habitat, and migration flows between neighboring patches are created asymmetrically, using one-way barriers such us funnels^41^. Most realized topologies are simple, e.g., forming two-dimensional grids. Our work is the first to show that even such simple structures can lead to strong amplification by controlling the migration rates between patches.

Amplifiers of natural selection could become important tools for in vitro evolution^42–45^, because they can greatly boost the ability to find advantageous mutants. Amplifiers could aid the discovery of optimized protein or nucleotide sequences for any medical or industrial purpose. They could also be a highly sensitive diagnostic tool for screening populations of replicating cells or viruses for the presence of faster growing (pathological) variants. Amplifiers should be especially useful in situations where the rate-limiting step is the discovery and evaluation of marginally advantageous mutants.

Some naturally occurring population structures could be amplifiers of natural selection. For example, the germinal centers of the immune system might constitute amplifiers for the affinity maturation process of adaptive immunity^46^. Habitats of animals that are divided into multiple islands with a central breeding location could potentially also act as amplifiers of selection. Our theory helps to identify those structures in natural settings.

Our study also establishes structural features that are necessary for amplification. For example, under temperature initialization, amplification cannot arise from the topology alone, but crucially depends on the migration rates between neighboring sites. Similarly, any search for natural amplifiers must focus on structures where the effect of self-loops is present, meaning that the offspring of reproducing individuals occasionally remains local and does not migrate to neighboring locations.

Most of the research in the field, including the current work, has focused on the populations that evolve according to the standard birth–death updating. An interesting direction for future research is to study if similar results can be achieved for death–birth updating.

## Methods

Here we describe the basic model and outline the mathematical methods. Further details are given in Supplementary Note 1.

### Model

Population structure is captured by a graph *G* with *N* vertices and directed edges that are possibly weighted and include self-loops. The vertices represent individuals, the edges represent interactions (Fig. 1). The weight of an edge between vertices *i* and *j* is denoted by $w_{ij}$, and captures the rate at which vertex *i* interacts with *j*. Alternatively, the rates can be captured by allowing multiple edges between vertices, in which case the structure is modeled by an unweighted multigraph. The degree of a vertex is the number of edges adjacent to it.

Individuals are of two types: residents with fitness 1 and (advantageous) mutants with fitness *r* > 1. The fitness of individual occupying vertex *i* is denoted by *f*_*i*_.

The population evolves according to birth–death updating. In each step, one individual is chosen for reproduction randomly and proportionally to its fitness, and then one of the adjacent edges is chosen randomly and proportionally to the edge weight. The selected individual produces a copy of itself (birth) and sends this copy along the selected edge to replace the individual at the other end of the edge (death). That is, individual *i* is selected for reproduction with probability $f_i/\mathop {\sum}\nolimits_j f_j$ and its adjacent edge $(i,i{\prime})$ is then selected with probability $w_{ii{\prime}}/\mathop {\sum}\nolimits_j w_{ij}$. The individual at vertex *i*′ then becomes the same type as individual at vertex *i*. Note that due to different degrees and weights, an edge between individuals *i* and $i{\prime}$ can be used more frequently in direction “from *i* to $i{\prime}$” than in direction “from $i{\prime}$ to $i$”.

The state of the process is given by vector $(x_1,x_2, \ldots ,x_N)$, where *x*_*i*_ = 1 represents that the individual occupying vertex *i* is a mutant and *x*_*i*_ = 0 for a resident. The process starts with a single mutant on one vertex and stops either when all the individuals become mutants (fixation) or residents (extinction) (Fig. 1a).

### Initialization scheme

In the beginning, the position of the single mutant can be chosen either uniformly at random (uniform initialization, *U*) or proportionally to the temperature of the vertex (temperature initialization, *T*). The temperature *t*_*h*_ of vertex *h* is defined as

1 $$t_h = \mathop {\sum}\limits_i \frac{{w_{ih}}}{{\mathop {\sum}\limits_j w_{ij}}}$$

and corresponds to how frequently each vertex is being replaced by reproduction events happening in the neighboring vertices.

### Amplifiers

The probability of fixation for a single mutant with relative fitness *r* appearing on graph *G* according to initialization scheme *U* (or *T*) is denoted by $\rho (G,r,U)$ (or $\rho (G,r,T)$).

Denoting by *K*_*N*_ the complete graph on *N* vertices, we have

2 $$\rho \left( {K_N,r,U} \right) = \rho \left( {K_N,r,T} \right) = \frac{{1 - 1{\mathrm{/}}r}}{{1 - 1{\mathrm{/}}r^N}} \to 1 - 1{\mathrm{/}}r$$

as $N \to \infty$. Graphs for which the fixation probability is greater than this for any *r* > 1 are called unif-amplifiers or temp-amplifiers based on the initialization scheme.

The most well-known unif-amplifiers are Star graphs *S*_*N*_ (see Fig. 1d) for which $\rho (S_N,r,U) \to 1 - 1/r^2 > 1 - 1/r$ as $N \to \infty$. However, Star graphs are not temp-amplifiers, since $\rho (S_N,r,T) \to 0$.

We are interested in the behavior for large population sizes. A sequence of graphs $G_1,G_2, \ldots$ of increasing size is called a strong unif-amplifier if, in the limit, the fixation probability under uniform initialization tends to 1 for arbitrary, fixed *r* > 1 (that is, if for any *r* > 1, we have $\mathop {{\rm{lim}}}\nolimits_{i \to \infty } \rho (G_i,r,U) \to 1$). Strong temp-amplifiers are defined analogously, requiring that $\mathop {{\rm{lim}}}\nolimits_{i \to \infty } \rho (G_i,r,T) \to 1$.

### Fundamental questions

Despite the rich interest in amplifiers of natural selection, many basic questions have remained unanswered. The fundamental open questions are the following.

First, are there strong amplifiers for temperature initialization? More generally, are there population structures that function as strong amplifiers for both uniform and temperature initialization?

Second, similarly to the Star, does there exist a graph without self-loops and/or without weights that is an amplifier for temperature initialization, achieving fixation probability at least 1 − 1/*r*^2^ for large *N*?

Third, are there simple structures, such as graphs with bounded degree, that are strong amplifiers for uniform initialization?

### Construction of strong amplifiers

In our positive result, we answer the first fundamental question by proving that almost every family of graphs of increasing population size can be turned into a strong amplifier (both strong unif-amplifier and strong temp-amplifier) by allowing self-loops and assigning weights to edges. Self-loops are natural. They indicate that the offspring can replace the parent^25^. The standard Moran process is given by a complete graph with self-loops.

In the proof^47^, we start by defining a subset of vertices called a hub and partitioning the remaining vertices into subsets called branches in such a way that each branch connects to the hub. The partitioning has the property that the hub is larger than each branch individually, but smaller than all of them combined. Such a partitioning is possible for all graphs that have diameter polynomially smaller than *N* (i.e., the distance between every pair of nodes is at most $N^{1 - \varepsilon }$, where $\varepsilon \,> \,0$ is fixed and independent of *N*).

Once we construct the partitioning with the required properties, we proceed by assigning weights in such a way that, intuitively, (i) in each branch, there is a sense of global flow directed toward the hub; and (ii) the hub is isothermal and evolves faster than the rest of the graph. The success of the construction then relies on the following two principles.

**Fig. 5:**
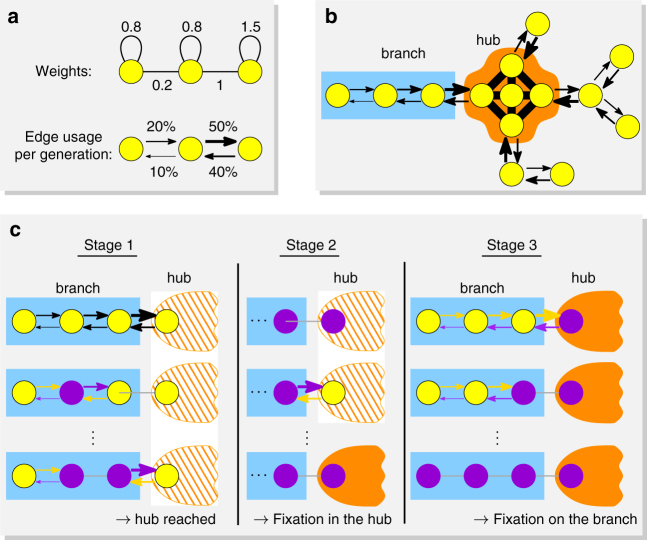
Details of steps to fixation. **a** Assigning different weights to edges and self-loops changes the frequency with which each edge is used in each direction. Thicker arrows indicate edges that are used more frequently. **b** Our weight assignment creates a global sense of flow in the branches, directed toward the hub. The hub itself is almost isothermal and evolves fast. **c** Three stages to fixation illustrated on a single branch and the connecting vertex in the hub. After fixating on the hub at the end of Stage 2 (hub becomes dark orange), mutants spread to all the branches and fixate on the whole graph

First, the weights create a sense of global flow. The weight assignment in the branches guarantees that every edge is used more frequently in the direction toward the hub than in the direction away from the hub. Moreover, by assigning suitable weights to the self-loops, we achieve that edges closer to the hub are used more frequently then edges further away from the hub. See Fig. 5a for a small numerical illustration. These two facts imply that a mutant arising in a branch will propagate toward the hub and repeatedly try to invade it.

Second, there is an important asymmetry between mutants and residents on a well-mixed population. For large population size *N*, the fixation probability of a single mutant with fitness *r* > 1 invading a well-mixed population of *N* residents tends to the positive constant 1 − 1/*r*. On the other hand, the probability that a single resident takes over a large well-mixed population of advantageous mutants is ~*r*^−*N*^, i.e., exponentially small in *N*. The weight assignment within the hub makes the hub behave approximately like a well-mixed population. Therefore, once the mutants fixate in the hub, they are extremely likely to resist the upcoming invasion attempts of the residents.

With these two principles in mind, we can informally argue as follows. Since the hub occupies only a small portion of the graph, the first mutant most likely appears in some branch. We focus on that branch and the hub (see Fig. 5b) and prove that due to the biased flow toward the hub, the mutants spread all the way to the hub (see Fig. 5c, Stage 1). Once in the hub, the mutants have a constant chance to fixate there. If the first invading mutant lineage fails in the hub, another such lineage will be generated from the branch. Eventually, the mutants take over the hub (see Fig. 5c, Stage 2). From that point on, it is extremely unlikely that the residents could win the hub back. In order to fixate on the branch, the mutants have to proceed against the natural direction of the flow, which is, in absolute terms, fairly improbable. However, the alternative of residents taking over the hub is much more improbable. Thus, with high probability, the mutants will succeed in fixating on the branch (see Fig. 5c, Stage 3). Similarly, they fixate on all the other branches.

For details, see Supplementary Note 1, Section 5, and the references therein.

### Necessary conditions for amplification

In our negative results, we answer the second and the third fundamental question by proving that both self-loops and weighted edges are essential for existence of strong amplifiers.

First, in order to address the second fundamental question, we consider temperature initialization on an arbitrary graph.

In order to prove that self-loops are necessary features for strong amplification, we consider a graph without self-loops (possibly with weighted edges). Then, given any possible starting position *i* of the mutant, we find that the probability, *p*_*i*_, that the mutant is replaced by one of its resident neighbors before it reproduces even once equals $p_i = t_i/(t_i + r)$, where *t*_*i*_ is the temperature of vertex *i*. Therefore, the fixation probability starting from a state with single mutant at vertex *i* is at most $1 - t_i/(t_i + r)$. Taking all possible starting positions into account, we establish an upper bound $1 - 1/(r + 1)$ on the fixation probability using Cauchy–Schwarz inequality. This implies that without self-loops, strong amplification under temperature initialization is not possible.

In order to prove that weighted edges are also necessary, we consider an unweighted graph (possibly with self-loops). We argue similarly and establish an upper bound $1 - 1/(4r + 2)$ on the fixation probability. Thus, the second fundamental question is answered in negative.

Second, in order to address the third fundamental question, we consider uniform initialization on graphs with bounded degree. As above, we first consider a graph without self-loops (possibly with weighted edges). Given any such graph *G*, we single out a subset *V*^*h*^ of vertices with high temperature that we call hot vertices. Formally, *V*^*h*^ consists of vertices that are replaced by at least one of their neighbors with rate at least 1/*c*, where *c* is the constant that bounds the degree. We prove that the subset *V*^*h*^ is large (namely, $\left| {V^h} \right| \ge N{\mathrm{/}}c$) and that the fixation probability starting from a single hot vertex is small (namely, smaller than $rc{\mathrm{/}}(1 + rc)$). Accounting of all hot vertices, we establish that $\rho (G,r,U) \le 1 - 1{\mathrm{/}}(c + cr^2)$. The case of unweighted graphs (that possibly have self-loops) follows similarly, by noticing that under bounded degree, all vertices are sufficiently hot. The bound we obtain is $\rho (G,r,U) \le 1 - 1{\mathrm{/}}(1 + rc)$. Altogether, this answers the third fundamental question in negative. For details, see Supplementary Note 1, Section 4, and the references therein.

### Data and code availability

The data sets generated and analyzed during the current study and the related computer code are available in the Figshare^48^ repository, 10.6084/m9.figshare.6323240.v1.

## Electronic supplementary material

Supplementary Information
