# Supplementary material for: Construction of arbitrarily strong amplifiers of natural selection using evolutionary graph theory
Source: Commun Biol. 2018 Jun 14;1:71. doi: 10.1038/s42003-018-0078-7 (PMC6123726; doi:10.1038/s42003-018-0078-7)
Supplement: Supplementary file 1 — Supplementary Information [file 42003_2018_78_MOESM1_ESM.pdf]

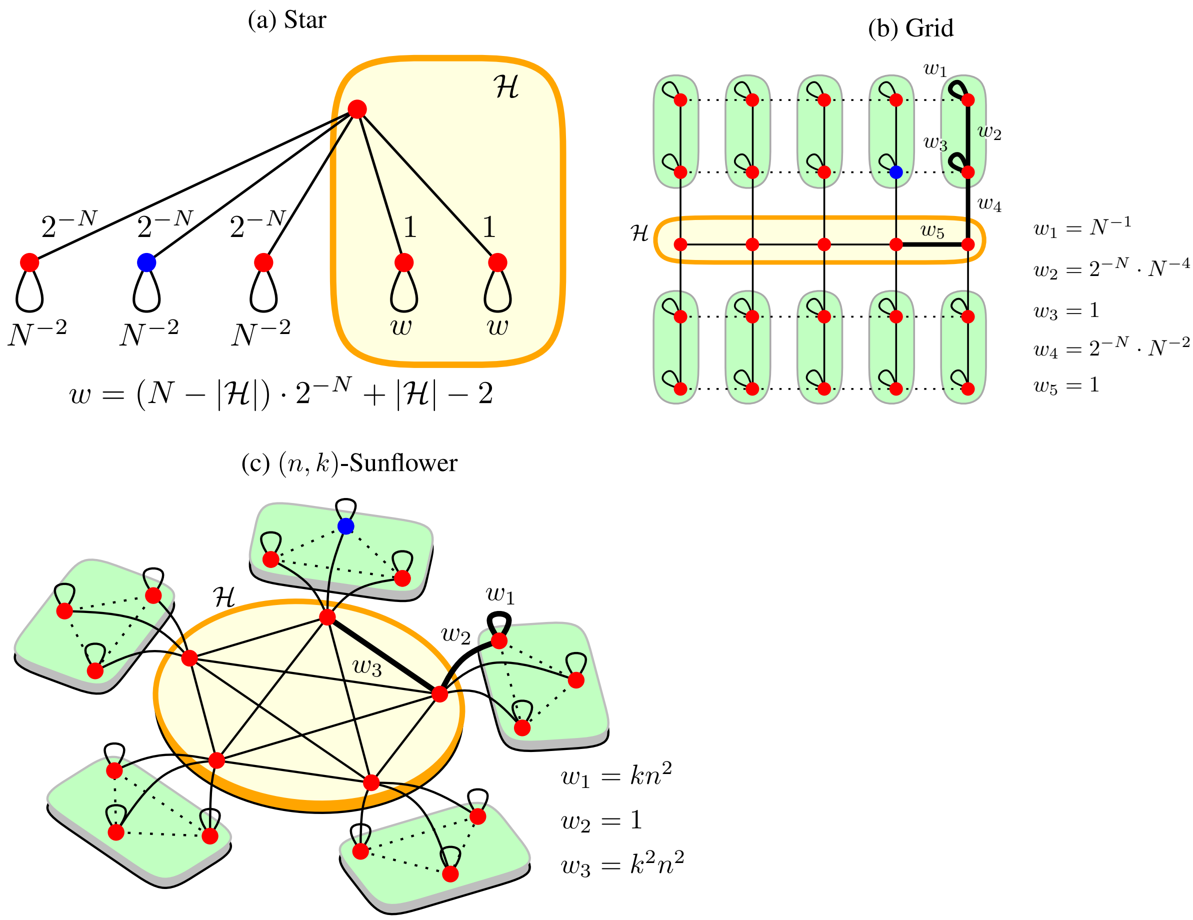

Supplementary Figure 1. Explicit examples of weight assignment. For several explicit graph families (Stars, Grids, Sunflowers), we present a way to partition them into a hub and branches and to assign weights to edges and self-loops to obtain strong amplifiers. See Supplementary Notes, Section 6.2 for details.

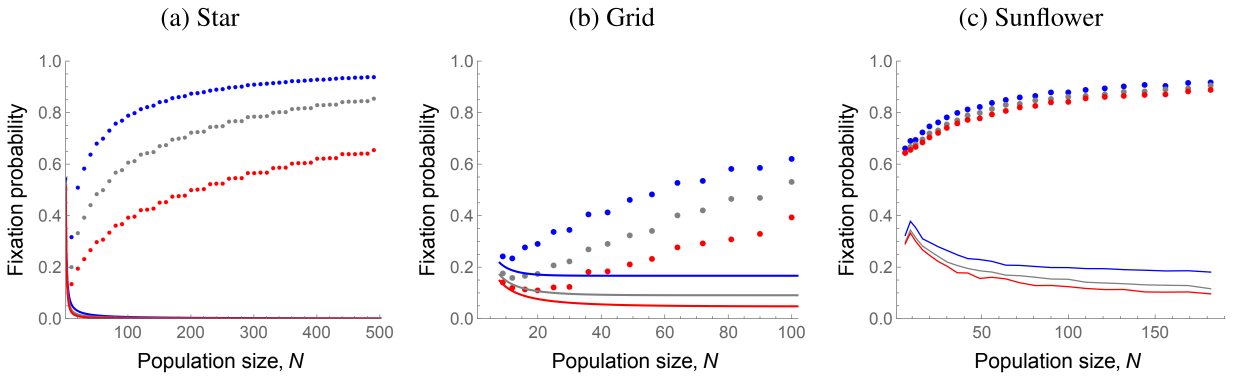

Supplementary Figure 2. Almost any topology can be turned into a strong amplifier under temperature initialization. Similarly to Fig. 3 from the main article, we consider Stars, Grids, and Sunflowers, this time under temperature initialization. For each graph family, we compare the fixation probability of the unweighted (lines) and the weighted version (dots) as function of the population size, N, for temperature initialization.

	Temperature		Uniform*	
	Loops	No Loops	Loops	No Loops
Weights	✓	$\times$	✓	$\times$
No Weights	$\times$	$\times$	$\times$	$\times$

Supplementary Table 1. Summary of our results on existence of strong amplifiers. We consider different initialization schemes (temperature initialization or uniform initialization) and graph families (presence or absence of loops and/or weights). The "✓" symbol marks that for given choice of initialization scheme and graph family, almost all graphs admit a weight function that makes them strong amplifiers. The "$\times$" symbol marks that for given choice of initialization scheme and graph family, no strong amplifiers exist (under any weight function). The asterisk signifies that the negative results under uniform initialization only hold for bounded degree graphs.

Supplementary Note 1: Construction of arbitrarily strong amplifiers of natural selection using evolutionary graph theory

Andreas Pavlogiannis[1*†], Josef Tkadlec[1*], Krishnendu Chatterjee[1], Martin A. Nowak[2]

[1]IST Austria, A-3400 Klosterneuburg, Austria
[2]Program for Evolutionary Dynamics, Department of Organismic and Evolutionary Biology, Department of Mathematics, Harvard University, Cambridge, MA 02138, USA

[*]These authors contributed equally to this work.
[†]To whom correspondence should be addressed; E-mail: pavlogiannis@ist.ac.at.

Contents

1 Organization **4**

2 Model and Summary of Results **5**
2.1 Model 5
2.2 Results 8

3 Preliminaries: Formal Notation **10**
3.1 The Moran Process on Weighted Structured Populations 10
3.2 Initialization and Fixation Probabilities 10
3.3 Strong Amplifier Graph Families 11

4 Negative Results **11**

5 Positive Result **12**

6 Explicit Examples **12**
6.1 Explicit Unweighted Graphs 12
6.2 Explicit Weight Assignments 13

7 Details of Simulation Results **14**

1 Organization

The organization of this document is as follows: In Section 2, we present the detailed description of our model and an overview of our results. Then we present the formal notation (Section 3) and we formally

state the negative results (Section 4) and the positive result (Section 5). Fully rigorous mathematical proofs, with all the technical details and complete calculations, are available in [21]. Here we focus on presenting the key ideas, omitting some of those details. Next we present several explicit examples of arbitrarily strong amplifiers (Section 6) and the detailed description of simulation results (Section 7).

2 Model and Summary of Results

2.1 Model

The birth-death Moran process. The *Moran process* considers a population of n individuals, which undergoes reproduction and death, and each individual is either a resident or a mutant [18]. The residents and the mutants have constant fitness 1 and r, respectively. The Moran process is a discrete-time stochastic process defined as follows: in the initial step, a single mutant is introduced into a homogeneous resident population. At each step, an individual is chosen randomly for reproduction with probability proportional to its fitness; another individual is chosen uniformly at random for death and is replaced by a new individual of the same type as the reproducing individual. Eventually, this Markovian process ends when all individuals become of one of the two types. The probability of the event that all individuals become mutants is called the *fixation* probability.

The Moran process on graphs. In general, the Moran process takes place on a population structure, which is represented as a graph. The vertices of the graph represent individuals and edges represent interactions between individuals [14, 19]. Formally, let $G_n = (V_n, E_n, W_n)$ be a weighted, directed graph, where $V_n = \{1, 2, \ldots, n\}$ is the vertex set , E_n is the Boolean edge matrix, and W_n is a stochastic weight matrix. An edge is a pair of vertices (i, j) which is indicated by $E_n[i, j] = 1$ and denotes that there is an interaction from i to j (whereas we have $E_n[i, j] = 0$ if there is no interaction from i to j). The stochastic weight matrix W_n assigns weights to interactions, i.e., $W_n[i, j]$ is positive iff $E_n[i, j] = 1$, and for all i we have $\sum_j W_n[i, j] = 1$. For a vertex i, we denote by $\mathsf{In}(i) = \{j \mid E_n[j, i] = 1\}$ (resp., $\mathsf{Out}(i) = \{j \mid E_n[i, j] = 1\}$) the set of vertices that have incoming (resp., outgoing) interaction or edge to (resp., from) i. Similarly to the Moran process, at each step an individual is chosen randomly for reproduction with probability proportional to its fitness. An edge originating from the reproducing vertex is selected randomly with probability equal to its weight. The terminal vertex of the chosen edge takes on the type of the vertex at the origin of the edge. In other words, the stochastic matrix W_n is the weight matrix that represents the choice probability of the edges. We only consider graphs which are *connected*, i.e., every pair of vertices is connected by a path. This is a sufficient condition to ensure that in the long run, the Moran process reaches a homogeneous state (i.e., the population consists entirely of individuals of a single type). The well-mixed population is represented by a complete graph where all edges have equal weight of $1/n$.

Classification of graphs. We consider the following classification of graphs:

1. *Directed vs undirected graphs.* A graph $G_n = (V_n, E_n, W_n)$ is called *undirected* if for all $1 \leq i, j \leq n$ we have $E_n[i, j] = E_n[j, i]$. In other words, there is an edge from i to j iff there is an edge from j to i, which represents symmetric interaction. If a graph is not undirected, then it is called a *directed* graph.

2. *Self-loop free graphs.* A graph $G_n = (V_n, E_n, W_n)$ is called a *self-loop free* graph iff for all $1 \leq i \leq n$ we have $E_n[i,i] = W_n[i,i] = 0$.

3. *Weighted vs unweighted graphs.* A graph $G_n = (V_n, E_n, W_n)$ is called an *unweighted* graph if for all $1 \leq i \leq n$ we have

$$W_n[i,j] = \begin{cases} \frac{1}{|\mathsf{Out}(i)|} & j \in \mathsf{Out}(i); \\ 0 & j \notin \mathsf{Out}(i) \end{cases}$$

   In other words, in unweighted graphs for every vertex the edges are choosen uniformly at random. Note that for unweighted graphs the weight matrix is not relevant, and can be specified simply by the graph structure (V_n, E_n). In the sequel, we will represent unweighted graphs as $G_n = (V_n, E_n)$.

4. *Bounded degree graphs.* The degree of a graph $G_n = (V_n, E_n, W_n)$, denoted $\deg(G_n)$, is $\max\{\mathsf{In}(i), \mathsf{Out}(i) \mid 1 \leq i \leq n\}$, i.e., the maximum in-degree or out-degree. For a family of graphs $(G_n)_{n>0}$ we say that the family has bounded degree, if there exists a constant c such that the degree of all graphs in the family is at most c, i.e., for all n we have $\deg(G_n) \leq c$.

Initialization of the mutant. The fixation probability is affected by many different factors [20]. In a well-mixed population, the fixation probability depends on the population size n and the relative fitness advantage r of mutants [15, 19]. For the Moran process on graphs, the fixation probability also depends on the population structure, which breaks the symmetry and homogeneity of the well-mixed population [13, 12, 6, 14, 4, 7, 22, 10]. Finally, for general population structures, the fixation probability typically depends on the initial location of the mutant [2, 3], unlike the well-mixed population where the probability of the mutant fixing is independent of where the mutant arises [15, 19]. There are two standard ways mutants may arise in a population [14, 1]. First, mutants may arise spontaneously and with equal probability at any vertex of the population structure. In this case we consider that the mutant arise at any vertex uniformly at random and we call this *uniform initialization.* Second, mutants may be introduced through reproduction, and thus arise at a vertex with rate proportional to the incoming edge weights of the vertex. We call this *temperature initialization.* In general, uniform and temperature initialization result in different fixation probabilities.

Amplifiers, quadratic amplifiers, and strong amplifiers. Depending on the initialization, a population structure can distort fitness differences [14, 19, 4], where the well-mixed population serves as a canonical point of comparison. Intuitively, amplifiers of selection exaggerate variations in fitness by increasing (respectively decreasing) the chance of fitter (respectively weaker) mutants fixing compared to their chance of fixing in the well-mixed population. In a well-mixed population of size n, the fixation probability is

$$\frac{1 - 1/r}{1 - (1/r)^n}.$$

Thus, in the limit of large population (i.e., as $n \to \infty$) the fixation probability in a well-mixed population is $1 - 1/r$. We focus on two particular classes of amplifiers that are of special interest. A family of graphs $(G_n)_{n>0}$ is a *quadratic* amplifier if in the limit of large population the fixation probability is $1 - 1/r^2$. Thus, a mutant with a 10% fitness advantage over the resident has approximately the same chance of fixing in quadratic amplifiers as a mutant with a 21% fitness advantage in the well-mixed population. A family of graphs $(G_n)_{n>0}$ is an *arbitrarily strong* amplifier (hereinafter called simply a strong amplifier) if for any constant $r > 1$ the fixation probability approaches 1 at the limit of large population sizes, whereas when $r < 1$, the fixation probability approaches 0. There is a much finer classification of amplifiers presented

in [1]. We focus on quadratic amplifiers which are the most well-known among polynomial amplifiers, and strong amplifiers which represent the strongest form of amplification.

Remark 1. Amplifiers tend to have fixation times longer than the well mixed population. Therefore they are especially useful in situations where the rate limiting step is the discovery and evaluation of marginally advantageous mutants. An interesting direction for future work would be to consider amplifiers as well as the time-scale of evolutionary trajectories.

Existing results. We summarize the main existing results in terms of uniform and temperature initialization.

1. *Uniform initialization.* First, consider the family of Star graphs, which consist of one central vertex and $n-1$ leaf vertices, with each leaf being connected to and from the central vertex. Star graphs are unweighted, undirected, self-loop free graphs, whose degree is linear in the population size. Under uniform initialization, the family of Star graphs is a quadratic amplifier [14, 19]. A generalization of Star graphs, called Superstars [14, 19, 11, 5], are known to be strong amplifiers under uniform initialization [8]. The Superstar family consists of unweighted, self-loop free, but directed graphs where the degree is linear in the population size. Another family of directed graphs with strong amplification properties, called Megastars, was recently introduced in [8]. The Megastars are stronger amplifiers than the Superstars, as the fixation probability on the former is a approximately $1-n^{-1/2}$ (ignoring logarithmic factors), and is asymptotically optimal (again, ignoring logarithmic factors). In contrast, the fixation probability on the Superstars is approximately $1-n^{-1/2}$. In the limit of $n \to \infty$, both families approach the fixation probability 1.

2. *Temperature initialization.* While the family of Star graphs is a quadratic amplifier under uniform initialization, it is not even an amplifier under temperature initialization [1]. It was shown in [1] that by adding self-loops and weights to the edges of the Star graph, a graph family, namely the family of Looping Stars, can be constructed, which is a quadratic amplifier simultaneously under temperature and uniform initialization. Note that in contrast to Star graphs, the Looping Star graphs are weighted and also have self-loops.

Open questions. Despite several important existing results on amplifiers of selection, several basic questions have remained open:

1. *Question 1.* Does there exist a family of self-loop free graphs (weighted or unweighted) that is a quadratic amplifier under temperature initialization?

2. *Question 2.* Does there exist a family of unweighted graphs (with or without self-loops) that is a quadratic amplifier under temperature initialization?

3. *Question 3.* Does there exist a family of bounded degree self-loop free (weighted or unweighted) graphs that is a strong amplifier under uniform initialization?

4. *Question 4.* Does there exist a family of bounded degree unweighted graphs (with or without self-loops) that is a strong amplifier under uniform initialization?

5. *Question 5.* Does there exist a family of graphs that is a strong amplifier under temperature initialization? More generally, does there exist a family of graphs that is a strong amplifier both under temperature and uniform initialization?

To summarize, the open questions ask for (i) the existence of quadratic amplifiers under temperature initialization without the use of self-loops, or weights (Questions 1 and 2); (ii) the existence of strong amplifiers

under uniform initialization without the use of self-loops, or weights, and while the degree of the graph is small; and (iii) the existence of strong amplifiers under temperature initialization. While the answers to Question 1 and Question 2 are positive under uniform initialization, they have remained open under temperature initialization. Questions 3 and 4 are similar to 1 and 2, but focus on uniform rather than temperature initialization. The restriction on graphs of bounded degree is natural: large degree means that some individuals must have a lot of interactions, whereas graphs of bounded degree represent simple structures. Question 5 was mentioned as an open problem in [1]. Note that under temperature initialization, even the existence of a cubic amplifier, that achieves fixation probability at least $1 - (1/r^3)$ in the limit of large population, has been open [1].

2.2 Results

In this work we present several negative as well as positive results that answer the open questions (Questions 1-5) mentioned above. We first present our negative results.

Negative results. Our main negative results are as follows:

1. Our first result (Theorem 1) shows that for any self-loop free weighted graph $G_n = (V_n, E_n, W_n)$, for any $r \geq 1$, under temperature initialization the fixation probability is at most $1 - 1/(r + 1)$. The implication of the above result is that it answers Question 1 in negative.
2. Our second result (Theorem 2) shows that for any unweighted (with or without self-loops) graph $G_n = (V_n, E_n)$, for any $r \geq 1$, under temperature initialization the fixation probability is at most $1 - 1/(4r + 2)$. The implication of the above result is that it answers Question 2 in negative.
3. Our third result (Theorem 3) shows that for any bounded degree self-loop free graph (possibly weighted) $G_n = (V_n, E_n, W_n)$, for any $r \geq 1$, under uniform initialization the fixation probability is at most $1 - 1/(c + c^2 r)$, where c is the bound on the degree, i.e., $\deg(G_n) \leq c$. The implication of the above result is that it answers Question 3 in negative.
4. Our fourth result (Theorem 4) shows that for any unweighted, bounded degree graph (with or without self-loops) $G_n = (V_n, E_n)$, for any $r \geq 1$, under uniform initialization the fixation probability is at most $1 - 1/(1 + rc)$, where c is the bound on the degree, i.e., $\deg(G_n) \leq c$. The implication of the above result is that it answers Question 4 in negative.

Significance of the negative results. We now discuss the significance of the above results.

1. The first two negative results show that in order to obtain quadratic amplifiers under temperature initialization, self-loops and weights are inevitable, complementing the existing results of [1]. More importantly, it shows a sharp contrast between temperature and uniform initialization: while self-loop free, unweighted graphs (namely, Star graphs) are quadratic amplifiers under uniform initialization, no such graph families are quadratic amplifiers under temperature initialization.
2. The third and fourth results show that without using self-loops and weights, bounded degree graphs cannot be made strong amplifiers even under uniform initialization. See also Remark 4.

Positive result. Our main positive result shows the following:

1. For any constant $\epsilon > 0$, consider any connected unweighted graph $G_n = (V_n, E_n)$ of n vertices with self-loops and which has *diameter* at most $n^{1-\epsilon}$. The diameter of a connected graph is the maximum, among all pairs of vertices, of the length of the shortest path between that pair. We establish (Theorem 5) that there is a stochastic weight matrix W_n such that for any $r > 1$ the fixation probability on $G_n = (V_n, E_n, W_n)$ both under uniform and temperature initialization is at least $1 - \frac{1}{n^{\epsilon/3}}$. An immediate consequence of our result is the following: for any family of connected unweighted graphs with self-loops $(G_n = (V_n, E_n))_{n>0}$ such that the diameter of G_n is at most $n^{1-\epsilon}$, for a constant $\epsilon > 0$, one can construct a stochastic weight matrix W_n such that the resulting family $(G_n = (V_n, E_n, W_n))_{n>0}$ of weighted graphs is a strong amplifier simultaneously under uniform and temperature initialization. Thus we answer Question 5 in affirmative.

Significance of the positive result. We highlight some important aspects of the results established in this work.

1. First, note that for the fixation probability of the Moran process on graphs to be well defined, a necessary and sufficient condition is that the graph is connected. A uniformly chosen random connected unweighted graph of n vertices has diameter bounded by a constant, with high probability. Hence, within the family of connected, unweighted graphs, the family of graphs of diameter at most $O(n^{1-\epsilon})$, for any constant $0 < \epsilon < 1$, has probability measure 1. Our results establish a strong dichotomy: (a) the negative results state that without self-loops and/or without weights, *no* family of graphs can be a quadratic amplifier (even more so a strong amplifier) even for only temperature initialization; and (b) in contrast, for *almost all* families of connected graphs with self-loops, there exist weight functions such that the resulting family of weighted graphs is a strong amplifier both under temperature and uniform initialization.

2. Second, with the use of self-loops and weights, even simple graph structures, such as Star graphs, Grids, and well-mixed structures (i.e., complete graphs) can be made strong amplifiers.

3. Third, our positive result is constructive, rather than existential. In other words, we not only show the existence of strong amplifiers, but present a construction of them.

Our results are summarized in Supplementary Table 1.

Remark 2. *Edges with zero weight.* Note that edges can be effectively erased by being assigned zero weight. (However, no weight assignment can create edges that don't exist.) Therefore, when our construction works for some graph, it also works for a graph that contains some additional edges. In particular, our construction easily works for complete graphs. However, erasing edges is not necessary: the construction can be easily modified to a scenario in which each existing edge is assigned positive (that is, non-zero) weight.

Remark 3. *Relation between theoretical and simulation results.* Strong amplifiers can only exist in the limit of large population size (see Theorem 5). In Section 7 we show that our construction and weight assignment significantly increases the fixation probability even for graphs with small population size.

3 Preliminaries: Formal Notation

3.1 The Moran Process on Weighted Structured Populations

We consider a population of n individuals on a graph $G_n = (V_n, E_n, W_n)$. Each individual of the population is either a *resident*, or a *mutant*. Mutants are associated with a *reproductive rate* (or *fitness*) r, whereas the reproductive rate of residents is normalized to 1. Typically we consider the case where $r > 1$, i.e., mutants are *advantageous*, whereas when $r < 1$ we call the mutants *disadvantageous*. We now introduce the formal notation related to the process.

Configuration. A *configuration* of G_n is a subset $S \subseteq V$ which specifies the vertices of G_n that are occupied by mutants and thus the remaining vertices $V \setminus S$ are occupied by residents. We denote by $\mathsf{F}(S) = r \cdot |S| + n - |S|$ the total fitness of the population in configuration S, where $|S|$ is the number of mutants in S.

The Moran process. The birth-death Moran process on G_n is a discrete-time Markovian random process. We denote by X_i the random variable for a configuration at time step i, and $\mathsf{F}(\mathsf{X}_i)$ and $|\mathsf{X}_i|$ denote the total fitness and the number of mutants of the corresponding configuration, respectively. The probability distribution for the next configuration X_{i+1} at time $i + 1$ is determined by the following two events in succession:

Birth: One individual is chosen at random to reproduce, with probability proportional to its fitness. That is, the probability to reproduce is $r/\mathsf{F}(\mathsf{X}_i)$ for a mutant, and $1/\mathsf{F}(\mathsf{X}_i)$ for a resident. Let u be the vertex occupied by the reproducing individual.

Death: A neighboring vertex $v \in \mathsf{Out}(u)$ is chosen randomly with probability $W_n[u, v]$. The individual occupying v dies, and the reproducing individual places a copy of its own on v. Hence, if $u \in \mathsf{X}_i$, then $\mathsf{X}_{i+1} = \mathsf{X}_i \cup \{v\}$, otherwise $\mathsf{X}_{i+1} = \mathsf{X}_i \setminus \{v\}$.

The above process is known as the *birth-death* Moran process, where the death event is conditioned on the birth event, and the dying individual is a neighbor of the reproducing one.

Probability measure. Given a graph G_n and the fitness r, the birth-death Moran process defines a probability measure on sequences of configurations, which we denote as $\mathbb{P}^{G_n,r}[\cdot]$. If the initial configuration is $\{u\}$, then we define the probability measure as $\mathbb{P}_u^{G_n,r}[\cdot]$, and if the graph and fitness r is clear from the context, then we drop the superscript.

Fixation event. The fixation event, denoted $\mathcal{E}$, represents that all vertices are mutants, i.e., $\mathsf{X}_i = V$ for some i. In particular, $\mathbb{P}_u^{G_n,r}[\mathcal{E}]$ denotes the fixation probability in G_n for fitness r of the mutant, when the initial mutant is placed on vertex u. We will denote this fixation probability as $\rho(G_n, r, u) = \mathbb{P}_u^{G_n,r}[\mathcal{E}]$.

3.2 Initialization and Fixation Probabilities

We will consider three types of initialization, namely, (a) uniform initialization, where the mutant arises at vertices with uniform probability, (b) temperature initialization, where the mutant arises at vertices proportional to the temperature, and (c) convex combination of the above two.

Temperature. For a weighted graph $G_n = (V_n, E_n, W_n)$, the temperature of a vertex u, denoted $\mathsf{T}(u)$, is $\sum_{v \in \mathsf{In}(u)} W_n[v, u]$, i.e., the sum of the incoming weights. Note that $\sum_{u \in V_n} \mathsf{T}(u) = n$, and a graph is *isothermal* iff $\mathsf{T}(u) = 1$ for all vertices u.

Fixation probabilities. We now define the fixation probabilities under different initialization.

1. *Uniform initialization.* The fixation probability under uniform initialization is

$$\rho(G_n, r, \mathsf{U}) = \sum_{u \in V_n} \frac{1}{n} \cdot \rho(G_n, r, u).$$

2. *Temperature initialization.* The fixation probability under temperature initialization is

$$\rho(G_n, r, \mathsf{T}) = \sum_{u \in V_n} \frac{\mathsf{T}(u)}{n} \cdot \rho(G_n, r, u).$$

3. *Convex initialization.* In *η-convex initialization*, where $\eta \in [0, 1]$, the initial mutant arises with probability $(1 - \eta)$ via uniform initialization, and with probability η via temperature initialization. The fixation probability is then

$$\rho(G_n, r, \eta) = (1 - \eta) \cdot \rho(G_n, r, \mathsf{U}) + \eta \cdot \rho(G_n, r, \mathsf{T}).$$

3.3 Strong Amplifier Graph Families

A *family* of graphs $\mathcal{G}$ is an infinite sequence of weighted graphs $\mathcal{G} = (G_n)_{n \in \mathbb{N}^+}$.

- *Strong amplifiers.* A family of graphs $\mathcal{G}$ is a *strong uniform amplifier* (resp. *strong temperature amplifier*, *strong convex amplifier*) if for every fixed $r_1 > 1$ and $r_2 < 1$ we have that

$$\liminf_{n \to \infty} \rho(G_n, r_1, Z) = 1 \qquad \text{and} \qquad \limsup_{n \to \infty} \rho(G_n, r_2, Z) = 0 \ ;$$

  where $Z = \mathsf{U}$ (resp., $Z = \mathsf{T}$, $Z = \eta$).

Intuitively, strong amplifiers ensures (a) fixation of advantageous mutants with probability 1 and (b) extinction of disadvantageous mutants with probability 1. In other words, strong amplifiers represent the strongest form of amplifiers possible.

4 Negative Results

In the current section we state our negative results, which show the nonexistence of strong amplifiers in the absence of either self-loops or weights. Fully rigorous mathematical proofs, with all the technical details and complete calculations, are available in [21].

Theorem 1. *For all self-loop free graphs G_n and for every $r \geq 1$ we have $\rho(G_n, r, \mathsf{T}) \leq 1 - 1/(r + 1)$.*

Corollary 1. *There exists no self-loop free family of graphs which is a strong temperature amplifier.*

Theorem 2. *For all unweighted graphs G_n and for every $r \geq 1$ we have $\rho(G_n, r, \mathsf{T}) \leq 1 - 1/(4r + 2)$.*

Corollary 2. *There exists no unweighted family of graphs which is a strong temperature amplifier.*

Theorem 3. *For all self-loop free graphs G_n with $c = \deg(G_n)$, and for every $r \geq 1$ we have $\rho(G_n, r, \mathsf{U}) \leq 1 - 1/(c + r \cdot c^2)$.*

Corollary 3. *There exists no self-loop free, bounded-degree family of graphs which is a strong uniform amplifier.*

Theorem 4. *For all unweighted graphs* G_n *with* $c = \deg(G_n)$*, and for every* $r \geq 1$ *we have* $\rho(G_n, r, \mathsf{U}) \leq 1 - 1/(1 + r \cdot c)$.

Corollary 4. *There exists no unweighted, bounded-degree family of graphs which is a strong uniform amplifier.*

Remark 4. Theorems 3 and 4 establish the nonexistence of strong amplification with bounded degree graphs. A relevant result can be found in [16], which establishes an upperbound of the fixation probability of mutants under uniform initialization on unweighted, undirected graphs. If the bounded degree restriction is relaxed to bounded average degree, then recent results show that strong amplifiers (called *sparse incubators*) exist [9].

5 Positive Result

In the previous section we showed that self-loops and weights are necessary for the existence of strong amplifiers. In this section we state our positive result, namely that every family of undirected graphs with self-loops and whose diameter is not "too large" can be made a strong amplifier by using appropriate weight functions W that assign non-negative real weights to edges and self-loops of the graphs. Fully rigorous mathematical proofs, with all the technical details and complete calculations, are available in [21].

Theorem 5. *Let* $\varepsilon > 0$ *and* $n_0 > 0$ *be any two fixed constants, and consider any sequence of unweighted, undirected graphs* $(G_n)_{n>0}$ *such that* $\operatorname{diam}(G_n) \leq n^{1-\varepsilon}$ *for all* $n > n_0$*. There exists a sequence of weight functions* $(\mathsf{w}_n)_{n>0}$ *such that the graph family* $\mathcal{G} = (G_n^{\mathsf{w}_n})$ *is a (i) strong uniform, (ii) strong temperature, and (iii) strong convex amplifier.*

6 Explicit Examples

In this section we present explicit examples of unweighted graph families and our weight assignment that turns them into arbitrarily strong amplifiers.

6.1 Explicit Unweighted Graphs

Complete graph K_N**.**

- Population size: N.
- Edges: Every vertex is connected to every vertex (including self-loops).

Star graph S_N**.**

- Population size: $N = 1 + (N - 1)$.
- Vertices: One vertex in the center, $N - 1$ vertices (*leafs*) around it.
- Edges: The center is connected to all the leafs.

Grid graph $G_{a,b}$.

- Population size: $N = a \cdot b$.
- Vertices: Aligned in a grid-like fashion with a rows and b columns.
- Edges: Every vertex is connected to four surrounding vertices (one above, below, to the left, and to the right). In order to avoid boundary conditions, the grid "wraps around", i.e. the vertices in the first row are connected to the vertices in the last row and the same holds for columns.

$(n, k_1, \dots, k_n)$-Sunflower graph and (n, k)-Sunflower graph.

- Population size: $N = n + (k_1 + \dots + k_n)$.
- Vertices: A complete graph of size n in the center, and n surrounding *petals* which are complete graphs of sizes $k_1, \dots, k_n$, respectively.
- Edges: In addition to the edges within center and petals, every petal is connected with all its vertices to a unique vertex from the center.
- When $k_1 = \dots = k_n = k$ we obtain so-called (n, k)-Sunflower.

Remark 5. Note that Grids are graphs of bounded degree – any vertex has only four neighbors (five including a self-loop), no matter how large the Grid is.

6.2 Explicit Weight Assignments

For several explicit graph families, we present a way to partition them into hub and branches and to assign weights to edges and self-loops to obtain strong amplifiers.

Star graph S_N: $N \to \infty$.

- Hub and branches: The hub $\mathcal{H}$ consists of the center vertex together with $\lfloor\sqrt{N}\rfloor$ other leafs called *hub-leafs*. Every other leaf is a single branch.
- Weights:

$$\mathsf{w}(u,u) = \begin{cases} 0 & \text{if } u \text{ is the center,} \\ (N - |\mathcal{H}|) \cdot 2^{-N} + |\mathcal{H}| - 2 & \text{if } u \text{ is a hub-leaf,} \\ N^{-2} & \text{if } u \text{ is outside the hub,} \end{cases} \qquad \mathsf{w}(u,v) = \begin{cases} 1 & \text{if } u, v \in \mathcal{H}, \\ 2^{-N} & \text{otherwise.} \end{cases}$$

See Supplementary Fig. 1(a) for an illustration.

Grid graph $G_{n,n}$: $n \to \infty$.

- Hub and branches: The hub $\mathcal{H}$ is the middle row (or one of the two middle rows if n is even), the rest is split into branches by assigning weight 0 to all the remaining horizontal edges.
- Weights: Recall that $N = n^2$ and $\lambda(u)$ is the distance from vertex u to the hub.

$$\mathsf{w}(u,u) = \begin{cases} 0 & \text{if } u \in \mathcal{H}, \\ N^{-(l-1)} & \text{if } \lambda(u) = l, \end{cases} \qquad \mathsf{w}(u,v) = \begin{cases} 1 & \text{if } u, v \in \mathcal{H}, \\ 2^{-N} \cdot N^{-2l} & \text{if } \lambda(u) + 1 = \lambda(v) = l. \end{cases}$$

See Supplementary Fig. 1(b) for an illustration.

(n,k)**-Sunflower graph:** $n, k \to \infty$**.**

- Hub and branches: The hub $\mathcal{H}$ is the complete graph in the center. Every other vertex is a single branch.
- Weights:

$$\mathsf{w}(u,u) = \begin{cases} 0 & \text{if } u \in \mathcal{H}, \\ kn^2 & \text{otherwise,} \end{cases} \qquad \mathsf{w}(u,v) = \begin{cases} k^2 n^2 & \text{if } u,v \in \mathcal{H}, \\ 0 & \text{if } u,v \notin \mathcal{H}, \\ 1 & \text{otherwise.} \end{cases}$$

See Supplementary Fig. 1(c) for an illustration.

Remark 6. Note that $n \times n$ Grid graphs are bounded degree graphs and our construction turns them into strong amplifiers.

7 Details of Simulation Results

Here we present details of the computer simulation results showed in Fig. 3 of main article.

Fig. 3 of main article shows how simple structures can be turned into strong amplifiers under uniform initialization by assigning weights according to our algorithm (see Section 6.2). Unless stated otherwise, the values plotted are obtained by simulating the process 10 000 times. For completeness, in Supplementary Fig. 2 we also present analogous comparisons for temperature initialization.

Fig. 3(A). We consider Star graphs S_N with $N = 10, 20, \ldots, 500$. For unweighted Star, the exact fixation probability under both uniform and temperature initialization follows from formula in [17]. The values for weighted Star were computed numerically by solving large systems of linear equations.

Fig. 3(B). We consider $n \times n$ and $n \times (n+1)$ Grid graphs of sizes $N = 9, 12, 16, 20, \ldots, 100$. Unweighted grid with N vertices is isothermal so the fixation probability under both uniform and temperature initialization is given by $(1 - 1/r)/(1 - 1/r^N)$.

Fig. 3(C). We consider $(n, n-1)$ and $(n, n-2)$-Sunflower graphs of sizes $N = 6, 9, 12, 16, \ldots, 182$.

Supplementary References

[1] B. Adlam, K. Chatterjee, and M. A. Nowak. Amplifiers of selection. *Proceedings of the Royal Society of London A: Mathematical, Physical and Engineering Sciences*, 471(2181), 2015.

[2] B. Allen, C. Sample, Y. A. Dementieva, R. C. Medeiros, C. Paoletti, and M. A. Nowak. The molecular clock of neutral evolution can be accelerated or slowed by asymmetric spatial structure. page 31, Sept. 2014.

[3] T. Antal, S. Redner, and V. Sood. Evolutionary dynamics on degree-heterogeneous graphs. *Phys. Rev. Lett.*, 96:188104, 2006.

[4] M. Broom and J. Rychtář. An analysis of the fixation probability of a mutant on special classes of non-directed graphs. *Proc. R. Soc. A Math. Phys. Eng. Sci.*, 464(2098):2609–2627, Oct. 2008.

[5] J. Díaz, L. A. Goldberg, G. B. Mertzios, D. Richerby, M. Serna, and P. G. Spirakis. Approximating fixation probabilities in the generalized moran process. *Algorithmica*, 69(1):78–91, 2014.

[6] R. Durrett and S. A. Levin. Stochastic spatial models: a user's guide to ecological applications. *Philos. Trans. R. Soc. London. Ser. B Biol. Sci.*, 343(1305):329–350, 1994.

[7] M. Frean, P. B. Rainey, and A. Traulsen. The effect of population structure on the rate of evolution. *Proc. R. Soc. B Biol. Sci.*, 280(1762):20130211, July 2013.

[8] A. Galanis, A. Göbel, L. A. Goldberg, J. Lapinskas, and D. Richerby. Amplifiers for the moran process. *CoRR*, abs/1512.05632, 2015.

[9] L. A. Goldberg, J. Lapinskas, J. Lengler, F. Meier, K. Panagiotou, and P. Pfister. Asymptotically optimal amplifiers for the moran process.

[10] B. Houchmandzadeh and M. Vallade. The fixation probability of a beneficial mutation in a geographically structured population. *New J. Phys.*, 13:073020, July 2011.

[11] A. Jamieson-Lane and C. Hauert. Fixation probabilities on superstars, revisited and revised. *Journal of Theoretical Biology*, 382:44–56, 2015.

[12] S. A. Levin. Population dynamic models in heterogeneous environments. *Annu. Rev. Ecol. Syst.*, 7(1):287–310, 1976.

[13] S. A. Levin and R. T. Paine. Disturbance, patch formation, and community structure. *Proc. Natl. Acad. Sci.*, 71(7):2744–2747, 1974.

[14] E. Lieberman, C. Hauert, and M. A. Nowak. Evolutionary dynamics on graphs. *Nature*, 433(7023):312–316, 01 2005.

[15] T. Maruyama. A Markov process of gene frequency change in a geographically structured population. *Genetics*, 76(2):367–377, 1974.

[16] G. B. Mertzios, S. Nikoletseas, C. Raptopoulos, and P. G. Spirakis. Natural models for evolution on networks. *Theor. Comput. Sci.*, 477:76–95, 2013.

[17] T. Monk, P. Green, and M. Paulin. Martingales and fixation probabilities of evolutionary graphs. *Proc. R. Soc. A Math. Phys. Eng. Sci.*, 470(2165):20130730, 2014.

[18] P. A. P. Moran. *The statistical processes of evolutionary theory*. Oxford University Press, Oxford, England, 1962.

[19] M. Nowak. *Evolutionary Dynamics*. Harvard University Press, 2006.

[20] Z. Patwa and L. M. Wahl. The fixation probability of beneficial mutations. *J. R. Soc. Interface*, 5(28):1279–1289, Nov. 2008.

[21] A. Pavlogiannis, J. Tkadlec, K. Chatterjee, and M. A. Nowak. Strong amplifiers of natural selection: Proofs. *arXiv preprint arXiv:1802.02509*, 2018.

[22] M. Whitlock. Fixation probability and time in subdivided populations. *Genetics*, 779(June):767–779, 2003.
